# Effect of Medicaid expansion on cancer treatment and survival among Medicaid beneficiaries and the uninsured

**DOI:** 10.1002/cam4.7461

**Published:** 2024-07-06

**Authors:** Kristin M. Primm, Hui Zhao, Naomi N. Adjei, Charlotte C. Sun, Alen Haas, Larissa A. Meyer, Shine Chang

**Affiliations:** ^1^ Department of Epidemiology The University of Texas MD Anderson Cancer Center Houston Texas USA; ^2^ Department of Epidemiology and Biostatistics The University of California San Francisco San Francisco California USA; ^3^ Department of Health Services Research The University of Texas MD Anderson Cancer Center Houston Texas USA; ^4^ Department of Gynecologic Oncology and Reproductive Medicine The University of Texas MD Anderson Cancer Center Houston Texas USA

**Keywords:** breast cancer, colorectal cancer, epidemiology and prevention, non small cell lung cancer, medicaid expansion, survival

## Abstract

**Background:**

The Affordable Care Act expanded Medicaid coverage for people with low income in the United States. Expanded insurance coverage could promote more timely access to cancer treatment, which could improve overall survival (OS), yet the long‐term effects of Medicaid expansion (ME) remain unknown. We evaluated whether ME was associated with improved timely treatment initiation (TTI) and 3‐year OS among patients with breast, cervical, colon, and lung cancers who were affected by the policy.

**Methods:**

Medicaid‐insured or uninsured patients aged 40–64 with stage I–III breast, cervical, colon, or non‐small cell lung cancer within the National Cancer Database (NCDB). A difference‐in‐differences (DID) approach was used to compare changes in TTI (within 60 days) and 3‐year OS between patients in ME states versus nonexpansion (NE) states before (2010–2013) and after (2015–2018) ME. Adjusted DID estimates for TTI and 3‐year OS were calculated using multivariable linear regression and Cox proportional hazards regression models, respectively.

**Results:**

ME was associated with a relative increase in TTI within 60 days for breast (DID = 4.6; *p* < 0.001), cervical (DID = 5.0 *p* = 0.013), and colon (DID = 4.0, *p* = 0.008), but not lung cancer (*p* = 0.505). In Cox regression analysis, ME was associated with improved 3‐year OS for breast (DID hazard ratio [HR] = 0.82, *p* = 0.009), cervical (DID‐HR = 0.81, *p* = 0.048), and lung (DID‐HR = 0.87, *p* = 0.003). Changes in 3‐year OS for colon cancer were not statistically different between ME and NE states (DID‐HR, 0.77; *p* = 0.075).

**Conclusions:**

Findings suggest that expanded insurance coverage can improve treatment and survival outcomes among low income and uninsured patients with cancer. As the debate surrounding ME continues nationwide, our findings serve as valuable insights to inform the development of policies aimed at fostering accessible and affordable healthcare for all.

## 1 INTRODUCTION

Medicaid expansion (ME) was a key provision of the Affordable Care Act (ACA) that allowed states the option to extend Medicaid eligibility to individuals with incomes up to 138% of the federal poverty level.^1^, ^2^ Unlike other ACA provisions that were implemented nationally, the decision to adopt ME was left to individual states. This variation in states' adoption of ME provides a natural experiment to evaluate the impact of insurance expansion on access to care and health outcomes.

In the United States, health insurance coverage is a critical determinant of access to care and outcomes among individuals with cancer. Uninsured individuals face significant barriers to care at every stage of the cancer care continuum, from screening and diagnosis to treatment, survivorship, and palliative care.^3^, ^4^ Research has demonstrated that patients without insurance are more likely to experience treatment delays, are more likely to be diagnosed with advanced‐stage disease, and have poorer survival outcomes compared to those with private insurance coverage.^4^, ^5^, ^6^

Within oncology, studies across various cancer types have demonstrated a strong association between prolonged time to treatment and worse overall survival (OS).^7^, ^8^, ^9^, ^10^, ^11^, ^12^ Since its implementation, ME has been associated with improved access to primary and preventive care, improved access to screening, and earlier stage cancer diagnosis, and reduced mortality in the general population.^13^, ^14^, ^15^, ^16^, ^17^, ^18^, ^19^, ^20^ However, many existing studies include a large number of patients not targeted by ME (i.e., patients with private insurance, Medicare, or other governmental insurance). The ACA's ME sought to improve coverage for low‐income individuals without insurance, and therefore should have minimal impact on patients who are eligible for Medicare or covered by private insurance.^2^, ^21^ As such, the impact of ME on receipt of timely treatment, and other cancer‐specific outcomes such as survival among the target population of intended beneficiaries (i.e., Medicaid‐eligible individuals) remains unknown. This study investigated the impact of ME on receipt of timely treatment and OS in a target population of patients with common cancers who were most affected by the policy. We hypothesize that expanded Medicaid coverage will allow more people to gain access to screening and early detection services, leading to earlier diagnosis, earlier treatment initiation, and ultimately better OS for these patients.

## 2 METHODS

Data were obtained from the National Cancer Database (NCDB), a nationwide clinical oncology database. The NCDB is a joint project of the Commission on Cancer of the American College of Surgeons and the American Cancer Society. The NCDB includes patient‐level data from over 1500 Commission on Cancer accredited facilities, representing more than 70% of all newly diagnosed cancer cases nationwide.^3^, ^22^ All patient information in the NCDB is de‐identified, and therefore, considered exempt from human subjects review by The University of Texas MD Anderson Cancer Center Institutional Review Board.

### 2.1 Patient population

Patients with a confirmed diagnosis of first primary female breast, cervical, colon, or non‐small cell lung (referred to hereafter as simply lung) cancer from 2010 to 2018 were identified using International Classification of Diseases for Oncology, third edition site codes (Table S1).

Patients younger than age 40 were excluded due to suppression of data in the NCDB, and patients older than age 64 were excluded due to Medicare eligibility. We also excluded patients with noninvasive in‐situ tumors and those with stage IV disease, those with missing or unknown time to treatment, and those with missing follow‐up information. Patients were then selected based on their state ME status. In this study, ME states included 19 states that expanded Medicaid in January 2014. Non‐expansion states included 19 states that did not expanded Medicaid eligibility by the end of 2019. Patients residing in states that expanded Medicaid before or after January 2014 (i.e., early and late ME states) were excluded due to differences in the timing of policy adoption (Table S2). The year 2014 was excluded from analyses as it served as a washout/phase‐in period and the year 2019 was excluded due to missing follow‐up data. Finally, we limit our primary analysis specifically to Medicaid and uninsured patients to focus on the population most likely affected by expanded Medicaid eligibility. Sensitivity analysis with the inclusion of patients with private and governmental insurance was also performed.

### 2.2 Outcomes and covariates

Outcomes of interest were time to treatment initiation (TTI) and 3‐year OS. TTI was defined in the NCDB as the time in days from diagnosis to the receipt of the first cancer‐directed treatment (i.e., surgery, radiotherapy, or systemic therapy [chemotherapy, immunotherapy, or hormonal therapy]), and dichotomized based on whether the treatment was initiated within 60 days of cancer diagnosis (≤60 days vs. >60 days). Shorter TTI has been associated with better OS across several types of cancer.^7^, ^9^, ^10^, ^12^ The 60‐day treatment interval was selected as an indicator of timely (≤60 days) versus delayed (>60 days) access to healthcare. We conducted sensitivity analyses using alternate criteria of TTI within 30, 90, and 120 days and obtained similar results with each of these alternatives.

Three‐year OS was measured as the number of months from diagnosis until death or last contact, with patients surviving more than 3 years censored after 36 months. The 3‐year endpoint was selected to be similar to the median follow‐up time (36.9 months for individuals diagnosed in the post‐expansion period) (Table S3). We focused on OS because the NCDB does not contain information on cause of death, precluding analysis of cancer specific survival.

Clinical and demographic covariates included age group, sex (when applicable), race/ethnicity, Charlson–Deyo comorbidity score, zip code level median household income, residence in a metropolitan area, hospital transfer, and facility type.

### 2.3 Statistical analysis

A quasi‐experimental difference‐in‐differences (DID) approach was used to quantify the effect of ME on TTI within 60 days and 3‐year OS.^23^ In DID analyses, the primary independent variable was an interaction between state expansion status (ME states vs. NE states) and time period (2010–2013 vs. 2015–2018). Adjusted DID estimates for TTI and 3‐year OS were calculated using multivariable linear regression and Cox proportional hazards models, respectively. All DID models were adjusted for age, sex (when applicable), race and ethnicity, Charlson–Deyo score, residence in a metropolitan area, zip code level median income, primary site, hospital transfer, and facility type. The proportional hazards (PH) assumption was tested for each covariate using scaled Schoenfeld residuals; variables that violated the PH assumption were included as stratifying variables to allow for differing baseline hazards associated with these variables (Appendix S1). Kaplan–Meier survival curves by state expansion status and time period were also constructed.

A key aspect of the DID approach is the assumption of parallel trends between ME and NE states in the pre‐2014 period.^23^ The parallel trends assumption was first assessed with visual inspection of unadjusted trends and by conducting a falsification test by fitting a regression model for each outcome that included an interaction term between state expansion status and diagnosis year during the pre‐expansion period, as done in prior DID studies.^17^, ^19^, ^20^, ^24^, ^25^, ^26^, ^27^, ^28^, ^29^ Year by expansion group interaction terms for TTI outcomes were not statistically significant for nearly all cancer types except for breast cancer. No violations of parallel trends were observed for 3‐year OS (Tables S4 and S5). To address potential nonparallel trends for breast cancer TTI, additional adjustment for pre‐expansion time trends were included in the DID model for breast cancer TTI, as done in prior studies.^20^, ^26^

Several additional sensitivity analyses were conducted. First, we conducted sensitivity analyses using TTI within 30, 90, and 120 days as alternate measures of timely treatment. Second, we expand our analyses to include patients with private, Medicare, or other governmental insurance. Third, given the 3‐year follow up for survival analysis, patients diagnosed in 2013 living in ME states could experience ME during follow‐up. Therefore, we performed an additional sensitivity check for survival outcomes by excluding patients diagnosed in the year 2013. All statistical tests were two‐sided with statistical significance level at 0.05. All statistical analysis was performed using STATA/SE, version 18.0 (Stata Corp LLC, College Station, TX).^30^

## 3 RESULTS

Of the 76,504 patients diagnosed with stage I–III breast, cervical, colon, or lung cancer from 2010 to 2013 and 2015 to 2018, 34,920 (45.6%) resided in ME states and 41,584 (54.4%) resided in NE states (Table 1). Compared with ME states, patients in NE states had higher percentage of low‐income (34.2% vs. 25%), Black (26.7% vs. 18.5%), and nonmetropolitan (19.3% vs. 15.5%) residents. Patients in ME states were more likely to be treated at an academic research center, while patients in NE states were more likely to be treated at comprehensive community cancer centers. The proportion of stage I tumors increased from pre to post periods in both ME (from 43.0% to 50.9%) and NE (from 37.3% to 41.0%).

**TABLE 1:** Patient characteristics by State Medicaid Expansion Status and time period (*N* = 76,504).

Characteristic, *n* (%)	Pre‐expansion (2010–2013)		Post expansion (2015–2019)	
	Expansion states *N* = 15,959	Nonexpansion states *N* = 22,091	Expansion states *N* = 18,961	Nonexpansion states *N* = 19,493
Primary cancer site				
Breast	9623 (60.3)	12,941 (58.6)	11,929 (62.9)	11,959 (61.4)
Cervix	1369 (8.6)	2134 (9.7)	1346 (7.1)	1832 (9.4)
Colon	1289 (8.1)	2000 (9.1)	946 (5.0)	978 (5.0)
Lung	3678 (23)	5016 (22.7)	4740 (25)	4724 (24.2)
Sex				
Male	2565 (16.1)	3803 (17.2)	2867 (15.1)	3079 (15.8)
Female	13,394 (83.9)	18,288 (82.8)	16,094 (84.9)	16,414 (84.2)
Race				
Asian/Pacific Islander	944 (5.9)	460 (2.1)	1373 (7.2)	430 (2.2)
Black	3074 (19.3)	5913 (26.8)	3389 (17.9)	5186 (26.6)
Hispanic	1882 (11.8)	2809 (12.7)	2562 (13.5)	3237 (16.6)
Other	787 (4.9)	817 (3.7)	770 (4.1)	593 (3.0)
White	9272 (58.1)	12,092 (54.7)	10,867 (57.3)	10,047 (51.5)
Age				
40–49	5012 (31.4)	7025 (31.8)	5250 (27.7)	5860 (30.1)
50–59	7339 (46.0)	10,266 (46.5)	8909 (47.0)	9169 (47.0)
60–64	3608 (22.6)	4800 (21.7)	4802 (25.3)	4464 (22.9)
Charlson Comorbidity Index score				
0	11,874 (74.4)	16,303 (73.8)	14,235 (75.1)	14,479 (74.3)
1	2988 (18.7)	4434 (20.1)	3241 (17.1)	3423 (17.6)
≥2	1097 (6.9)	1354 (6.1)	1485 (7.8)	1591 (8.2)
AJCC clinical stage				
I	6860 (43.0)	8232 (37.3)	9660 (50.9)	8001 (41.0)
II	5121 (32.1)	7576 (34.3)	5322 (28.1)	6259 (32.1)
III	3978 (24.9)	6283 (28.4)	3979 (21.0)	5233 (26.8)
Area of residence				
Metropolitan	13,098 (82.1)	17,547 (79.4)	15,835 (83.5)	15,625 (80.2)
Nonmetropolitan	2526 (15.8)	4281 (19.4)	2884 (15.2)	3730 (19.1)
Unknown	335 (2.1)	263 (1.2)	242 (1.3)	138 (0.7)
Zip code‐level median income				
<$40,227	4023 (25.2)	7646 (34.6)	4694 (24.8)	6584 (33.8)
$40,227–$50,353	3477 (21.8)	5683 (25.7)	4086 (21.5)	4769 (24.5)
$50,354–$63,332	3065 (19.2)	3703 (16.8)	3870 (20.4)	3034 (15.6)
<$63,332	3435 (21.5)	2703 (12.2)	4014 (21.2)	2347 (12.0)
Unknown				
Facility type	1959 (12.3)	2356 (10.7)	2297 (12.1)	2759 (14.2)
Community cancer program	1596 (10.0)	1512 (6.8)	1784 (9.4)	1187 (6.1)
Comprehensive community cancer program	4287 (26.9)	8576 (38.8)	4833 (25.5)	7373 (37.8)
Academic research program	7165 (44.9)	7609 (34.4)	8999 (47.5)	7263 (37.3)
Integrated network cancer program	2911 (18.2)	4394 (19.9)	3345 (17.6)	3670 (18.8)
Insurance status				
Medicaid	1142 (76.1)	17,342 (91.5)	13,301 (60.2)	12,118 (62.2)
No insurance	3817 (23.9)	1619 (8.5)	8790 (39.8)	7375 (37.8)

### 3.1 Expansion associated effects on timely treatment initiation

**FIGURE 1:**
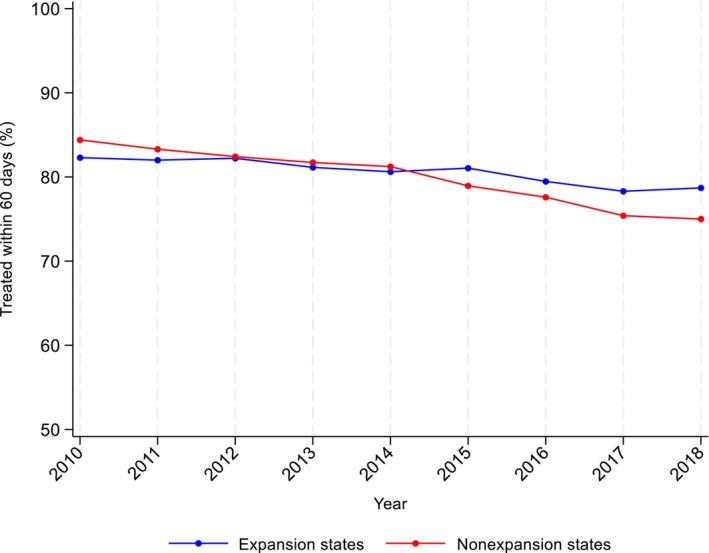
Unadjusted trends in the percentage of patients treated within 60 days by state expansion status.

In ME states, TTI within 60 days of diagnosis decreased over time from 81.7% before expansion to 79.3% after expansion (difference = −2.4%) (Figure 1; Table 2). In NE states, TTI within 60 days also declined from 82.9% to 76.8% following expansion (difference = −6.3%). Although TTI within 60 days decreased over time in both groups, declines were smaller in ME states, resulting in a relative increase in the percentage treated within 60 days compared with NE states (DID = 3.8, 95% CI = 2.6–4.9) after expansion.

**TABLE 2:** Changes in the percentage of patients treated within 60 days by state expansion status.

	Expansion states (*N* = 34,920)			Nonexpansion states (*N* = 41,584)			Adjusted DID^a^ (95% CI)	*p*‐value
	Pre‐2014, %	Post‐2014, %	Unadjusted difference (95% CI)	Pre‐2014, %	Post‐2014, %	Unadjusted difference (95% CI)		
All sites combined	81.7	79.3	−2.4 (−3.3 to −1.6)	82.9	76.8	−6.3 (−7.0 to −5.5)	3.8 (2.6–4.9)	<0.001
Breast	82.1	81.7	−0.3 (−1.3 to 0.7)	83.1	78.3	−5.0 (−6.0 to −4.0)	4.6 (3.2–6.0)	<0.001
Cervix	80.7	78.7	−2.3 (−5.3 to 0.8)	81.5	74.5	−7.4 (−9.9 to −4.8)	5.0 (1.0–8.9)	0.013
Colon	92.3	90.9	−1.7 (−4.0 to 0.7)	94.6	88.9	−5.9 (−7.9 to −4.0)	4.0 (1.1–7.0)	0.008
Lung	77.5	71.3	−6.4 (−8.3 to −4.5)	78.3	71.4	−6.9 (−8.6 to −5.2)	0.9 (−1.6 to 3.5)	0.505

*Note*: Authors' analysis of the National Cancer Database.

Abbreviations: CI, confidence interval; DID, difference‐in‐difference.

^a^ Adjusted for age, sex (when applicable), race, ethnicity, Charlson–Deyo score, residence in a metropolitan area, zip code level median income, primary site, hospital transfer, and facility type.

**FIGURE 2:**
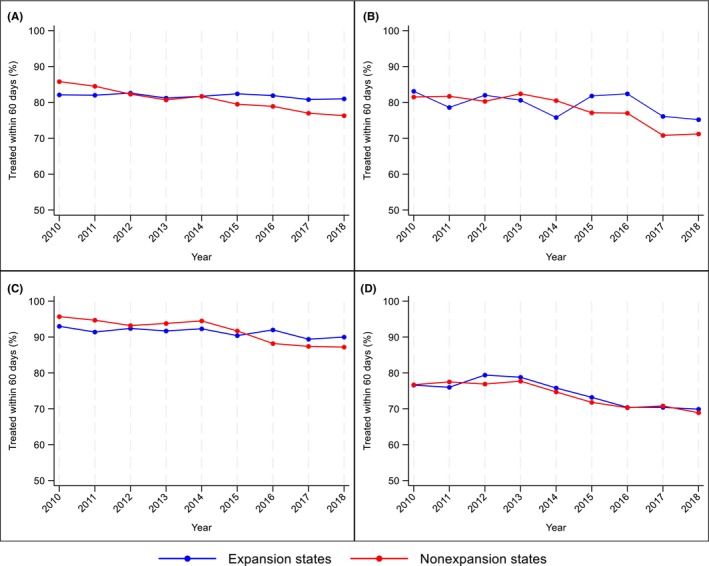
Unadjusted trends in the percentage of patients treated within 60 days by state expansion status and cancer site: (A) breast, (B) cervix, (C) colon, and (D) lung.

When examined by cancer type, similar changes in TTI were observed for most cancer types (Table 2; Figure 2). For breast, cervical, and colon cancer, the proportion of patients treated within 60 days did not change over time in ME states. In contrast, TTI within 60 days significantly decreased in NE states from pre to post periods for breast (difference = −5.0%), cervical (difference = −7.4%), and colon cancers (difference = −5.9%). As a result, DID estimates showed a relative increase in the percentage of patients treated within 60 days in ME states compared with NE states after 2014 for breast (DID = 4.6, 95% CI = 3.2–6.0), cervical (DID = 5.0, 95% CI = 1.0–8.9), and colon (DID = 4.0, 95% CI = 1.1–7.0) cancer. For lung cancer, the percentage of patients treated within 60 days of diagnosis decreased from pre to post periods in both ME (difference = −6.4%) and NE states (difference = −6.9%); however, the slope of decline was not statistically different between ME versus NE and states (DID = 0.9, 95% CI = −1.6 to 3.5).

### 3.2 Survival analysis

**FIGURE 3:**
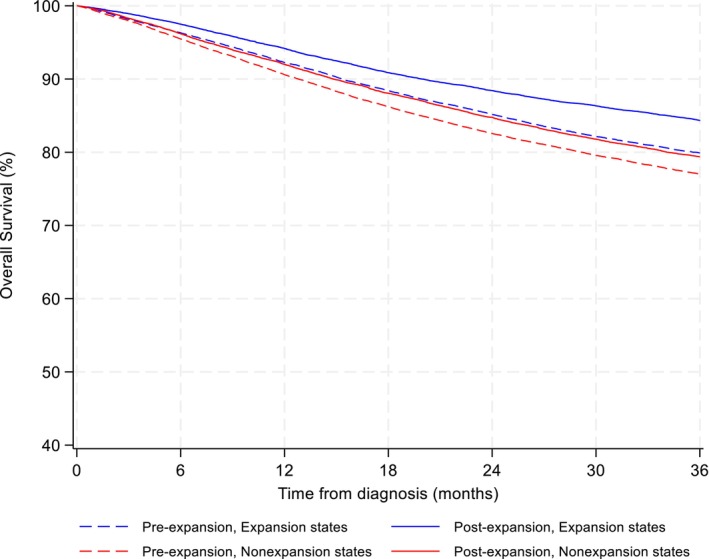
Unadjusted Kaplan–Meier survival curves for Medicaid expansion versus nonexpansion states in the pre‐expansion and post‐expansion period.

Kaplan–Meier survival curves show that patients in ME states generally experienced better survival compared to those living in NE states (Figure 3; Table S6). In multivariable Cox models, 3‐year OS improved from pre‐ to post‐2014 in both ME states (HR = 0.71, 95% CI = 0.67–0.75) and NE states (HR = 0.84, 95% CI = 0.80–0.88). In DID analysis, improvements in 3‐year OS were larger in ME states relative to NE states following expansion (DID‐HR = 0.84; 95% CI, 0.78–0.90; *p* < 0.001) (Table 3).

**TABLE 3:** Cox regression of 3‐year overall survival in expansion versus nonexpansion states.

	Expansion states (*N* = 34,920)		Nonexpansion states (*N* = 41,584)		Adjusted DID‐HR^b^ (95% CI)	*p*‐value
	Post vs. Pre HR^a^ (95% CI)	*p*‐value	Post vs. Pre HR^a^ (95% CI)	*p*‐value		
All cancers combined^c^	0.71 (0.67–0.75)	<0.001	0.84 (0.80–0.88)	<0.001	0.84 (0.78–0.90)	<0.001
Breast^d^	0.77 (0.68–0.87)	<0.001	0.93 (0.85–1.02)	0.116	0.82 (0.70–0.95)	0.009
Cervix^d^	0.86 (0.73–1.01)	0.081	1.09 (0.95–1.25)	0.239	0.81 (0.65–0.99)	0.048
Colon^d^	0.70 (0.60–0.91)	0.007	0.94 (0.79–1.12)	0.488	0.77 (0.60–1.03)	0.075
Lung^e^	0.67 (0.63–0.72)	0.001	0.76 (0.72–0.81)	<0.001	0.87 (0.79–0.95)	0.003

Abbreviations: CI, confidence interval; DID, difference‐in‐difference; HR, hazard ratio.

^a^ The pre/post Cox proportional hazards model compared the change in 3‐year OS in post‐2014 compared with pre‐2014 (the reference group). A HR <1 indicates an improvement in 3‐year OS in the post‐2014 study period compared with the pre‐2014 study period.

^b^ The DID‐HR refers to the ratio of pre/post HR in expansion states compared with the pre/post HR in nonexpansion states. A DID‐HR <1 indicates a greater improvement in expansion states compared with non‐expansion states.

^c^ Model adjusted for age, race, ethnicity, Charlson–Deyo score, residence in a metropolitan area, zip code level median income, hospital transfer, and facility type; with underlying stratification by cancer site and sex due to nonproportional hazards.

^d^ Model adjusted for age, sex (when applicable), race, ethnicity, Charlson–Deyo score, residence in a metropolitan area, zip code level median income, primary site, hospital transfer, and facility type.

^e^ Model adjusted for age, race, ethnicity, Charlson–Deyo score, metropolitan residence, zip code level median income, hospital transfer, and facility type; with underlying stratification by sex due to nonproportional hazards.

**FIGURE 4:**
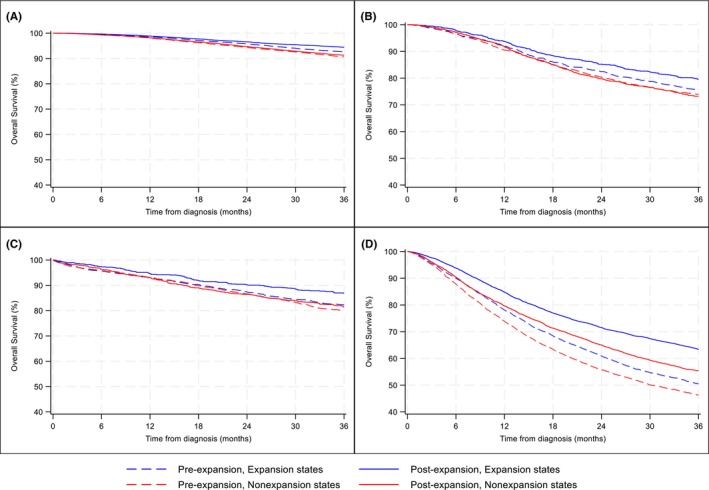
Unadjusted Kaplan–Meier survival curves for expansion versus nonexpansion states in the pre and post‐expansion periods by cancer site: (A) breast, (B) cervical, (C) colon, and (D) lung.

When stratified by cancer site, we observed consistent patterns, with ME states showing greater improvements in 3‐year OS over time compared with NE states for most cancer types (Table 3; Figure 4). Specifically, for breast and colon cancers, 3‐year OS improved from pre to post periods in ME states, whereas no statistically significant changes in 3‐year OS were observed in NE states. In DID analysis, ME was associated with improved 3‐year OS for breast (DID‐HR = 0.82, 95% CI = 0.70–0.95) and cervical (DID‐HR = 0.81; 95% CI = 0.65–0.99) in ME states compared with NE states; however, changes in 3‐year OS for colon cancer were not statistically different between ME and NE states (DID‐HR = 0.77; 95% CI, 0.60–1.03, *p* = 0.075). For lung cancer, although 3‐year OS improved in both ME (HR = 0.67, 95% CI = 0.63–0.72) and NE states (HR = 0.76, 95% CI = 0.72–0.81), the survival improvement was significantly greater in ME states relative to NE states (DID‐HR = 0.87, 95% CI = 0.79–0.95; *p* = 0.003).

### 3.3 Sensitivity analyses

In sensitivity analysis, we found similar results using TTI within 30 days (combined cancer sites, DID = 3.9, 95% CI = 2.5–5.2, *p* < 0.001), TTI within 90 days (combined cancer sites, DID = 1.9; 95% CI = 1.1–2.6, *p* < 0.001), and TTI within 120 days (combined cancer sites, DID = 1.0; 95% CI 0.4–1.5, *p* < 0.001) as alternative measures of timely treatment, although DID estimates were smaller for TTI within 90 days and TTI within 120 days (Table S7). In sensitivity analysis including patients with private and non‐Medicaid insurance types, DID estimates were considerably smaller for TTI within 60 days (DID = 1.1, 95% CI = 0.9–1.6) and 3‐year OS (DID‐HR = 0.94, 95% CI = 0.90–0.96), which is consistent with the treatment effects being diluted from the inclusion of individuals that are unaffected by ME (Tables S8 and S9). Sensitivity survival analyses excluding patients diagnosed in 2013 yielded similar results to our main analysis (Table S9).

## 4 DISCUSSION

Using a large nationwide database, we provide evidence that ME was associated with improved timely treatment and 3‐year survival among uninsured or Medicaid‐insured patients diagnosed with stage I–III breast, cervical, colon, or lung cancer. To the authors' knowledge, this study is one of the first to show a significant association between ME, timely treatment, and 3‐year OS specifically among Medicaid and uninsured patients. Our study adds to the growing literature highlighting the positive effects of ME on low‐income and medically underserved individuals with cancer.^21^, ^31^, ^32^, ^33^, ^34^

Results from this study demonstrate how improved insurance coverage can increase access to healthcare, resulting in more timely and effective treatment for low‐income and uninsured individuals. Although TTI has been increasing since the early 2000s, a major concern at the time of ME was that an influx of newly insured patients would lead to increased wait times and create additional delays in cancer care.^12^, ^20^ However, our TTI analysis revealed positive and significant DID estimates, indicating that the proportion of patients treated within 60 days decreased less in ME states compared to NE states after 2014. As a result, expansion states had a higher percentage of patients receiving treatment within 60 days than nonexpansion (NE) states in the post‐expansion period. Similar patterns in TTI were observed in site‐specific analysis; however, DID estimates for lung cancer TTI were not statistically significant. One possible explanation is the recent inclusion of genomic testing as part of the standard diagnostic evaluation for non‐small cell lung cancer, which has been associated with long turnaround times that could delay treatment.^35^, ^36^ Although genomic testing can improve lung cancer outcomes through personalized treatment, it introduces additional steps in the care process that could delay treatment initiation, regardless of state expansion status.^37^ This may partially explain the nonsignificant effect of ME on timely treatment for lung cancer. ME was also associated with improved 3‐year survival, especially for breast, cervical, and lung cancer patients. Although survival for colon cancer improved in both ME and NE states, the improvements were not statistically different between ME versus NE states.

Several studies have leveraged NCDB data to examine changes in TTI or OS following ME, with most showing modest or nonsignificant effects. For example, Takvorian et al. examined patients with breast, colon, and lung cancer and found no changes in TTI following ME.^20^ Another study of patients with cervical cancer also found no changes in timely treatment following ME.^19^ Other NCDB studies have found small improvements in OS associated with ME for patients with lung cancer, yet no effect on patients with breast, colon, or cervical cancer.^18^, ^19^, ^38^ Additional studies using other data sources (including SEER or NAACCR) have found small improvements in 2‐year OS for lung and colorectal cancer associated with ME, but no significant changes for patients with breast or cervical cancer.^32^, ^39^, ^40^ Although prior work has examined the association between ME, timely treatment, and cancer survival, most of these studies have included all insurance types in their study cohort, which could mask the impact of the policy on Medicaid eligible patients who are most likely to benefit from ME.^18^, ^19^, ^20^, ^32^, ^38^, ^41^ By focusing our analysis on Medicaid and uninsured patients, we provide a more granular and arguably more precise estimate of the policy's effect on its target population. In our analysis of Medicaid and uninsured patients, the DID estimates for TTI and OS are larger in magnitude compared to previous studies that included all insurance types in their analysis of ME.

The ACA's ME aimed to improve access to care for low‐income individuals without insurance, and therefore, should have minimal impact on patients who are eligible for Medicare or covered by private insurance. We show in sensitivity analyses that the inclusion of these groups can lead to an underestimation of true policy impact on patients most likely to benefit from ME. For example, when stratified by insurance type, the largest improvements in TTI and OS were observed among the uninsured, followed by Medicaid‐insured individuals, while changes for other insurance types were considerably smaller and mostly non‐significant. Improvements among the uninsured could be attributed to the fact that in most states, uninsured individuals diagnosed with cancer become eligible for Medicaid coverage after their diagnosis. Consequently, patients initially categorized as uninsured may have transitioned to Medicaid coverage shorty after diagnosis. However, because the NCDB only captures insurance status at diagnosis, we were unable to account for changes in insurance coverage over time.

In summary, ME can have a greater impact on Medicaid and uninsured patients compared to those with private insurance because it addresses some of the systemic barriers to care that these populations face. Expanded coverage benefits Medicaid and uninsured cancer patients by reducing financial barriers to care, enabling patients to access to a broader range of preventive health services, treatment options, and specialists that were previously unattainable due to financial constraints.^42^ This improved coverage enables more people to gain access to screenings, leading to earlier diagnosis and treatment of cancers and, ultimately, better survival. In contrast, privately insured patients already have consistent access to healthcare services, making the impact of ME on their timeliness of care and survival outcomes less pronounced. While the direct impact of ME might be less evident for those with non‐Medicaid coverage, the broader healthcare system improvements spurred by the ACA can indirectly lead to more efficient care delivery and improved outcomes for all patients.

Our study has several limitations. First, the NCDB exclusively includes data from CoC‐accredited institutions, which may not provide a complete representation of the entire population. CoC‐accredited hospitals may differ from non‐CoC‐accredited hospitals in terms of patient demographics, healthcare infrastructure, and treatment practices, potentially introducing selection biases that impact the generalizability of our findings.^22^, ^43^ Consequently, the data collected from the NCDB reflects only a subset of patients who underwent treatment within these facilities. It remains unclear how many low‐income patients were treated within these centers compared to facilities that did not contribute data to the NCDB. Also, the NCDB does not contain information on patients' state of residence, therefore, were unable to control for state‐level fixed effects in DID analyses. Furthermore, since the NCDB does not collect data on cause of death, we were unable to examine cause‐specific survival. Another limitation of this study is relatively short follow up after expansion. With 4 years of post‐expansion data, we were only able to examine expansion associated changes in 3‐year OS. It is also important to acknowledge that the survival outcomes might be influenced by lead time bias due to increased access to cancer screening. Finally, due to the observational nature of our study, results may be confounded by unobserved differences between patients in ME and NE states in the pre and post period.

## 5 CONCLUSION

In this study, ME was associated with smaller decrease in TTI and improved survival outcomes among patients treated at centers enrolled in NCDB. Findings suggest that expanded insurance coverage can improve timely treatment and survival outcomes among low income and uninsured patients with breast, cervical, colon, and lung cancer. As the ACA remains subject of debate across the United States, this study can help guide policies that promote accessible and affordable healthcare to address socioeconomic disparities associated with cancer outcomes.

## AUTHOR CONTRIBUTIONS

**Kristin M. Primm:** Conceptualization (lead); formal analysis (lead); investigation (lead); methodology (equal); visualization (lead); writing – original draft (lead); writing – review and editing (equal). **Hui Zhao:** Conceptualization (supporting); investigation (supporting); methodology (supporting); supervision (equal); writing – review and editing (equal). **Naomi N. Adjei:** Conceptualization (equal); writing – review and editing (equal). **Charlotte C. Sun:** Supervision (equal); writing – review and editing (equal). **Alen Haas:** Writing – review and editing (supporting). **Larissa A. Meyer:** Conceptualization (supporting); resources (supporting); supervision (equal); writing – review and editing (equal). **Shine Chang:** Funding acquisition (supporting); resources (supporting); supervision (supporting); writing – review and editing (equal).

## FUNDING INFORMATION

K.M.P is supported by the Cancer Prevention and Research Institute of Texas (CPRIT) Postdoctoral Fellowship in Cancer Prevention (RP 170259, Drs. Chang and Shete, PIs). N.N.A is supported by National Institutes of Health T32 Training Grant 5 T32 CA101642.

## CONFLICT OF INTEREST STATEMENT

All authors declare that they have no conflicts of interest.

## Supporting information


Data S1.

## Data Availability

This study utilized data from the 2020 NCDB Participant User File. The NCDB is available to investigators associated with CoC‐accredited cancer programs via an application process (https://www.facs.org/quality‐programs/cancer/ncdb/puf).
