# Supplementary material for: Effect of Medicaid expansion on cancer treatment and survival among Medicaid beneficiaries and the uninsured
Source: Cancer Med. 2024 Jul 6;13(13):e7461. doi: 10.1002/cam4.7461 (PMC11226780; doi:10.1002/cam4.7461)
Supplement: Supplementary file 1 — Data S1. [file CAM4-13-e7461-s001.docx]

SUPPLEMENTAL MATERIALS

**Supplementary Figure 1.** Cohort selection criteria

Excluded (n= 3,874,470)

- Diagnosed in 2014/washout period (n=440,662)
- Male breast cancer (n=15,853)
- Sequence number>1 (n=706,083)
- Not AJCC clinical stage I-III (n= 1,211,588)
- Age<40 (n= 76,856)
- Age>64 (n= 735,829)
- Early expansion states (n= 135,974)
- Late expansion states (n= 100,578)
- Non-Medicaid insurance (n=445,560)
- Missing/Unknown TTI (n=5,471)
- Missing/unknown survival information (n=16)

Met inclusion criteria for main analysis

(n=76,504)

Patients with breast, cervical, colon, or NSCLC in the NCDB PUF diagnosed from 2010-2018

(n=3,950,974)

**Supplementary Table 1.** International Classification of Diseases for Oncology third edition (ICD-O-3) codes used to identify cancer sites within the NCDB

| Site | ICD-O-3 code |
| --- | --- |
| Breast | C50.0-C50.9 |
| Cervix | C53.0-C53.9 |
| Colon | C18.0-C18.9 |
| Non-small cell lung | C34.0-34.9 (excluding histology codes 8041-8045) |

**Supplementary Table 2.** National Cancer Database Medicaid Expansion Status State Group variable definitions

| Definition | Included states |
| --- | --- |
| Non-expansion states | TN, NC, ID, GA, FL, MO, AL, MS, KS, TX, WI, UT, SC, SD, VA, OK, NE, WY, ME |
| 2014 Expansion states | KY, NV, CO, OR, NM, WV, AR, RI, AZ, MD, MA, ND, OH, IA, IL, VT, HI, NY, DE |
| Early Expansion states (2010-2013)* | WA, CA, NJ, MN, DC, CT |
| Late Expansion states (after Jan. 2014)* | NH, IN, MI, PA, AK, MT, LA |

*Early and late expansion states were excluded from our main analysis due to heterogenous timelines of policy adoption occurring before and after 2014

**Supplementary Table 3.** Mean and median follow-up (in months) by state expansion status and time period among Medicaid and uninsured patients aged 40-64 years.

| Characteristic | Expansion states | | Nonexpansion states | | Total | |
| --- | --- | --- | --- | --- | --- | --- |
|  | Pre-2014 | Post-2014 | Pre-2014 | Post-2014 | Pre-2014 | Post-2014 |
| Mean follow-up (SD) | 75.9 (40.5) | 43.8 (19.8) | 74.9 (41.6) | 42.3 (20.5) | 73.4 (41.1) | 43.0 (20.2) |
| Median follow-up (IQR) | 87.6 (70.4) | 44.6 (28.2) | 87.1 (74.4) | 43.0 (30.8) | 87.4 (72.7) | 43.8 (29.4) |

SD, Standard deviation, IQR, interquartile range.

**Appendix 1. Proportional hazard testing**

The proportional hazards (PH) assumption was tested for each covariate using scaled Schoenfeld residuals; variables that violated this assumption were included as stratifying variables in the final model to allow for differing baseline hazards associated with these variables. The PH assumption was tested for the overall (all cancers) model and separately by cancer site.

For the overall (all cancers combined) model, the PH assumption was violated for sex and cancer site; therefore, sex and cancer site were included as stratifying variables in the overall (all cancers) model. The cox model for lung cancer OS was also stratified by sex due to nonproportional hazards. No violations of the PH assumption were observed for the other cancer sites.

**Supplementary Table 4.** Parallel trends falsification test for treatment outcome: Interaction between state expansion status and diagnosis year during pre-2014 period.

| Characteristic | Adjusted^a^ DID  (95% CI) | P-value |
| --- | --- | --- |
| All sites combined | 0.75 (-0.03 to 1.52) | .060 |
| Breast | 1.50 (0.53 to 2.44) | .003 |
| Cervix | -0.03 (-2.60 to 2.54) | .980 |
| Colon | 0.15 (-1.37 to 1.67) | .850 |
| Lung | -0.66 (-2.29 to 0.96) | .422 |

DID, Difference-in-difference; CI, Confidence Interval

^a^ Adjusted difference-in-differences (DID) estimate represents the regression coefficient on an interaction term between expansion status and diagnosis year using data from the pre-2014 period only. Adjusted for age, sex (when applicable), race/ethnicity, income, residence in a metropolitan area, and facility type. DID estimates for TTI were derived using linear probability regression models.

**Supplementary Table 5.** Parallel trends test for 3-year OS: Interaction between state expansion status and diagnosis year during pre-2014 period.

| Characteristic | Adjusted^a^ DID-HR  (95% CI) | P-value |
| --- | --- | --- |
| All sites combined | 1.03 (0.99 - 1.07) | .154 |
| Breast | 1.00 (0.91 - 1.08) | .944 |
| Cervix | 0.99 (0.86 - 1.11) | .849 |
| Colon | 0.96 (0.82 – 1.12) | .621 |
| Lung | 1.06 (0.99 - 1.12) | .058 |

OS, overall survival; DID-HR, Difference-in-difference; HR, Hazards ratio; CI, Confidence Interval

^a^ Adjusted difference-in-differences (DID) estimate represents the regression coefficient on an interaction term between residence in an expansion state diagnosis year using data from the pre-expansion period only. Adjusted for age, sex (when applicable), race/ethnicity, comorbidity, income, residence in a metropolitan area, and facility type. Estimates for DID-HRs were derived using Cox-proportional hazards models.

**Supplementary Table 6.** Number of subjects (N) at risk by cancer site, state expansion group, and time from diagnosis in months

|  | Expansion states | | Nonexpansion states | |
| --- | --- | --- | --- | --- |
| Characteristic | Pre-2014  (N) | Post-2014  (N) | Pre-2014  (N) | Post-2014  (N) |
| All sites combined |  |  |  |  |
| 0 mo | 15,959 | 18,959 | 22,091 | 19,493 |
| 6 mo | 15,323 | 18,348 | 21,077 | 18,625 |
| 12 mo | 14,599 | 17,433 | 19,936 | 17,554 |
| 18 mo | 13,862 | 16,542 | 18,862 | 16,460 |
| 24 mo | 13,258 | 15,688 | 17,963 | 15,453 |
| 30 mo | 12,679 | 14,565 | 17,232 | 14,155 |
| 36 mo | 12,220 | 12,892 | 16,572 | 12,473 |
| Breast |  |  |  |  |
| 0 mo | 9,623 | 11,927 | 12,941 | 11,959 |
| 6 mo | 9,529 | 11,787 | 12,796 | 11,787 |
| 12 mo | 9,384 | 11,516 | 12,564 | 11,493 |
| 18 mo | 9,183 | 11,190 | 12,261 | 11,078 |
| 24 mo | 8,978 | 10,775 | 11,945 | 10,635 |
| 30 mo | 8,755 | 10,133 | 11,670 | 9,917 |
| 36 mo | 8,544 | 9,069 | 11,359 | 8,870 |
| Cervix |  |  |  |  |
| 0 mo | 1,369 | 1,346 | 2,134 | 1,832 |
| 6 mo | 1,306 | 1,282 | 2,052 | 1,748 |
| 12 mo | 1,230 | 1,189 | 1,915 | 1,605 |
| 18 mo | 1,120 | 1,089 | 1,768 | 1,445 |
| 24 mo | 1,056 | 1,017 | 1,655 | 1,298 |
| 30 mo | 985 | 926 | 1,560 | 1,148 |
| 36 mo | 935 | 817 | 1,488 | 974 |
| Colon |  |  |  |  |
| 0 mo | 1,289 | 946 | 2,000 | 978 |
| 6 mo | 1,218 | 902 | 1,903 | 924 |
| 12 mo | 1,171 | 865 | 1,844 | 883 |
| 18 mo | 1,127 | 823 | 1,773 | 821 |
| 24 mo | 1,081 | 785 | 1,700 | 776 |
| 30 mo | 1,032 | 733 | 1,619 | 717 |
| 36 mo | 996 | 634 | 1,545 | 627 |
| Lung |  |  |  |  |
| 0 mo | 3,678 | 4,740 | 5,016 | 4,724 |
| 6 mo | 3,270 | 4,377 | 4,326 | 4,166 |
| 12 mo | 2,814 | 3,863 | 3,613 | 3,573 |
| 18 mo | 2,432 | 3,440 | 3,060 | 3,116 |
| 24 mo | 2,143 | 3,111 | 2,663 | 2,744 |
| 30 mo | 1,907 | 2,773 | 2,383 | 2,373 |
| 36 mo | 1,745 | 2,372 | 2,180 | 2,002 |

**Supplementary Table 7.** Sensitivity analyses of Medicaid expansion associated changes in timely treatment initiation among Medicaid/Uninsured patients using alternative measures of timely treatment (N=76,504)

|  | Expansion states | | | Nonexpansion states | | |  |  |
| --- | --- | --- | --- | --- | --- | --- | --- | --- |
|  | Pre-  2014, % | Post-2014, % | Unadjusted difference  (95% CI) | Pre-  2014, % | Post-2014, % | Unadjusted difference  (95% CI) | Adjusted DID^a^  (95% CI) | p-value |
| TTI <30 days |  |  |  |  |  |  |  |  |
| All sites combined | 47.8 | 41.4 | -6.4 (-7.5 to -5.4) | 51.2 | 40.8 | -10.4 (-11.1 to -9.4) | 3.9 (2.5 to 5.2) | <.001 |
| Breast | 44.6 | 39.5 | -5.0 (-6.4 to -3.7) | 47.8 | 39.0 | -8.7 (-10.0 to -7.5) | 3.6 (1.8 to 5.4) | <.001 |
| Cervix | 47.7 | 42.2 | -5.5 (-0.9 to -1.7) | 48.5 | 36.5 | -11.9 (-14.9 to -8.8) | 7.1 (2.4 to 11.8) | .003 |
| Colon | 78.5 | 71.6 | -6.9 (-10.5 to -3.3) | 84.0 | 73.4 | -10.6 (-13.6 to -7.6) | 3.0 (-1.5 to 7.5) | .183 |
| Lung | 45.5 | 40.0 | -5.8 (-7.9 to -3.7) | 48.0 | 40.1 | -7.8 (-9.8 to -5.9) | 2.4 (-0.5 to 5.2) | .100 |
|  |  |  |  |  |  |  |  |  |
| TTI <90 days |  |  |  |  |  |  |  |  |
| All sites combined | 93.4 | 92.7 | -0.7 (-1.2 to -0.2) | 93.5 | 90.9 | -2.5 (-3.0 to -2.0) | 1.9 (1.1 to 2.6) | <.001 |
| Breast | 94.1 | 94.5 | 0.4 (-0.2 to 1.0) | 93.3 | 90.5 | -1.5 (-2.1 to -0.9) | 2.2 (1.3 to 3.1) | <.001 |
| Cervix | 93.6 | 92.5 | -1.1 (-3.0 to 0.) | 93.3 | 90.5 | -2.7 (-4.4 to 1.0) | 1.1 (-1.4 to 3.9) | .413 |
| Colon | 97.4 | 96.4 | -1.0 (-2.5 to 0.4) | 97.7 | 94.7 | -3.0 (-4.4 to -1.7) | 2.1 (0.1 to 4.0) | .040 |
| Lung | 90.1 | 87.5 | -2.5 (-3.9 to -1.1) | 90.6 | 86.5 | -4.1 (-5.3 to -2.8) | 1.6 (-0.3 to 3.5) | .094 |
|  |  |  |  |  |  |  |  |  |
| TTI <120 days |  |  |  |  |  |  |  |  |
| All sites combined | 96.8 | 96.7 | -0.1 (-0.1 to 0.2) | 97.1 | 95.9 | -1.2 (-1.5 to -0.8) | 1.0 (0.4 to 1.5) | <.001 |
| Breast | 97.2 | 97.8 | 0.4 (0.1 to 0.9) | 97.4 | 96.9 | -0.5 (-0.9 to -1.0) | 1.0 (0.4 to 1.6) | <.001 |
| Cervix | 97.6 | 95.8 | -1.8 (-3.1 to -0.4) | 97.3 | 95.8 | -1.4 (-2.6 to -0.3) | -0.4 (-2.1 to 1.4) | .686 |
| Colon | 98.8 | 98.3 | -0.4 (-1.4 to 0.6) | 98.9 | 98.2 | -0.7 (-1.6 to 0.2) | 0.4 (-1.0 to 1.8) | .472 |
| Lung | 94.9 | 94.0 | -0.9 (-1.9 to 0.1) | 95.3 | 92.8 | -2.5 (-3.4 to -1.6) | 1.8 (0.4 to 3.1) | .010 |

Abbreviations: DID, Difference-in-differences; CI, confidence interval; TTI, Time to treatment initiation

^a^ Adjusted for age, sex (when applicable), race/ethnicity, Charlson comorbidity score, zip code–level median income, primary site, residence in a metropolitan area, hospital transfer and facility type

**Supplementary Table 8.** Sensitivity analyses of Medicaid expansion associated changes in timely treatment initiation survival including patients with non-Medicaid insurance (N=497,073)

|  | Expansion states  N= 223,131 | | | Nonexpansion states  N 273,942 | | |  |  |
| --- | --- | --- | --- | --- | --- | --- | --- | --- |
|  | Pre-  2014, % | Post-2014, % | Unadjusted difference  (95% CI) | Pre-  2014, % | Post-2014, % | Unadjusted difference  (95% CI) | Adjusted DID^a^  (95% CI) | p-value |
| **TTI<60 days including patients with non-Medicaid insurance^b^** | | | | | | | | |
| All sites combined | 89.2 | 85.8 | -3.4 (-3.7 to -3.1) | 90.3 | 85.8 | -4.5 (-4.8 to -4.3) | 1.1 (0.9 to 1.6) | <.001 |
| Breast | 90.2 | 87.5 | -2.8 (-3.1 to -2.4) | 91.3 | 87.2 | -4.1 (-4.3 to -3.8) | 1.3 (0.9 to 1.7) | <.001 |
| Cervix | 86.3 | 83.5 | -2.8 (-4.4 to -1.2) | 87.1 | 82.1 | -4.9 (-6.3 to -3.5) | 2.1 (0.0 to 4.1) | .053 |
| Colon | 95.9 | 94.0 | -1.9 (-2.7 to -1.1) | 96.5 | 93.4 | -3.2 (-3.8 to -2.5) | 1.1 (0.1 to 2.1) | .040 |
| Lung | 82.4 | 77.0 | -5.5 (-6.3 to -4.7) | 84.0 | 77.8 | -6.2 (-6.9 to -5.4) | 0.9 (-0.1 to 2.0) | .090 |
|  |  |  |  |  |  |  |  |  |
| **TTI<60 days (all sites combined) stratified by insurance type** | | | | | | | | |
| Private | 91.4 | 88.2 | -3.2 (-3.5 to -2.9) | 92.7 | 88.2 | -4.5 (-4.7 to -4.2) | 1.2 (0.8 to 1.6) | .002 |
| Medicare | 83.9 | 79.4 | -4.5 (-5.6 to -3.4) | 86.5 | 81.1 | -5.4 (-6.3 to -4.5) | 1.0 (-0.4 to 2.4) | .153 |
| Other government | 85.2 | 81.8 | -3.4 (-6.2 to -0.6) | 86.2 | 81.5 | -4.7 (-6.4 to -2.9 | 0.3 (-2.9 to 3.4) | .876 |
| Medicaid | 81.2 | 79.1 | -2.1 (-3.0 to -1.1) | 83.6 | 77.8 | -5.8 (-6.9 to -4.9) | 3.8 (2.5 to 5.1) | <.001 |
| Uninsured | 83.5 | 81.5 | -4.1 (-4.2 to 0.2) | 81.9 | 74.9 | -7.0 (-8.2 to 5.7) | 5.2 (2.6 to 7.8) | <.001 |

^a^ Adjusted for age, sex (when applicable), race/ethnicity, Charlson comorbidity score, zip code–level median income, primary site, residence in a metropolitan area, hospital transfer and facility type

^b^ Includes patients with Private (n=364,057), Medicare (n=46,693), other governmental insurance (n=9,819) along with Medicaid (n=54,903) and uninsured (n=21,601) patients

**Supplementary Table 9**. Sensitivity analyses of Medicaid expansion associated changes in overall survival Including patients with non-Medicaid insurance (N=497,073)

|  | Expansion states  N= 223,131 | | Nonexpansion states  N= 273,942 | |  |  |
| --- | --- | --- | --- | --- | --- | --- |
|  | Post vs Pre HR^a^  (95% CI) | p-value | Post vs Pre HR^a^  (95% CI) | p-value | Adjusted DID-HR^b,c^  (95% CI) | p-value |
| **Exclude diagnosis year 2013** | | | | | | |
| All sites combined | 0.71 (0.66 - 0.76) | <.001 | 0.81 (0.77 - 0.85) | <.001 | 0.86 (0.80 - 0.93) | <.001 |
| Breast | 0.75 (0.67 - 0.86) | <.001 | 0.91 (0.83 – 0.99) | .047 | 0.82 (070 – 0.96) | .014 |
| Cervix | 0.93 (0.77 – 1.10) | .405 | 1.06 (0.92 – 1.23) | .422 | 0.89 (0.70 – 1.12) | .315 |
| Colon | 0.71 (0.56 - 0.91) | .012 | 0.95 (0.78 – 1.14) | .561 | 0.75 (0.55 – 1.03) | .080 |
| Lung | 0.66 (0.60 - 0.68) | <.001 | 0.72 (0.68 - 0.77) | <.001 | 0.90 (0.81 – 0.99) | .042 |
|  |  |  |  |  |  |  |
| **Including patients with non-Medicaid insurance^d^** | | | | | | |
| All sites combined | 0.75 (0.73 - 0.77) | <.001 | 0.84 (0.82 - 0.86) | <.001 | 0.94 (0.90 - 0.96) | .001 |
| Breast | 0.83 (0.78 - 0.87) | <.001 | 0.88 (0.84 – 0.92) | .001 | 0.94 (088 – 1.00) | .067 |
| Cervix | 0.85 (0.76 – 0.95) | .005 | 0.91 (0.83 – 0.99) | .030 | 0.94 (0.81 – 1.09) | .413 |
| Colon | 0.86 (0.76 - 0.96) | .003 | 0.88 (0.80 – 0.97) | <.001 | 0.91 (0.78 - 1.06) | .223 |
| Lung | 0.72 (0.70 - 0.75) | <.001 | 0.76 (0.73 - 0.78) | <.001 | 0.95 (0.91 – 1.00) | .040 |
|  |  |  |  |  |  |  |
| **Stratified by insurance type (All 4 cancer sites combined)** | | | | | | |
| Private | 0.75 (0.72-0.78) | <.001 | 0.82 (0.79-0.84) | <.001 | 0.93 (0.88-0.97) | .003 |
| Medicare | 0.77 (0.72-0.81) | <.001 | 0.80 (0.76-0.84) | <.001 | 0.95 (0.88-1.03) | .256 |
| Other government | 0.70 (0.57-0.86) | .001 | 0.78 (0.68-0.89) | <.001 | 0.90 (0.71-1.15) | .415 |
| Medicaid | 0.71 (0.67-0.75) | <.001 | 0.82 (0.77-0.87) | <.001 | 0.87 (0.80-0.94) | .001 |
| Uninsured | 0.76 (0.65-0.90) | <.001 | 0.89 (0.82-0.95) | .001 | 0.82 (0.69-0.98) | .031 |

Abbreviations: DID, Difference-in-differences; HR, Hazard ratio; CI, Confidence Interval

^a^ The pre/post Cox proportional hazards model compared the change in 3-year OS in post-2014 compared with pre-2014 (the reference group). A HR < 1 indicates an improvement in 3-year OS in the post-2014 study period compared with the pre-2014 study period

^b^ The DID-HR represents the ratio of pre/post HR in expansion states compared with the pre/post HR in nonexpansion states. DID-HR < 1 indicates a greater improvement in expansion states compared with non-expansion states.

^c^ Adjusted for age, sex (when applicable) race, ethnicity, Charlson-Deyo score, residence in a metropolitan area, zip code level median income, primary site, hospital transfer, and facility type

^d^ Includes patients with Private (n=364,057), Medicare (n=46,693), other governmental insurance (n=9,819) along with Medicaid (n=54,903) and uninsured (n=21,601) patients
